# Brain activity is not only for thinking

**DOI:** 10.1016/j.cobeha.2021.04.002

**Published:** 2021-05-12

**Authors:** Timothy O Laumann, Abraham Z Snyder

**Affiliations:** 1 Department of Psychiatry, Washington University in St. Louis School of Medicine, United States; 2 Department of Neurology, Washington University in St. Louis School of Medicine, United States; 3 Department of Radiology, Washington University in St. Louis School of Medicine, United States

## Abstract

The human brain is a complex organ with multiple competing imperatives. It must perceive and interpret the world, incorporate new information, and maintain its functional integrity over the lifespan. Neural activity is associated with all of these processes. Spontaneous BOLD signals have been invoked as representing neural activity associated with all of these processes. However, their exact role in these processes remains controversial. Here, we review learning machine theory, molecular mechanisms of synaptic plasticity and homeostasis, and recent experimental evidence to suggest that spontaneous BOLD activity may be more closely aligned with off-line plasticity and homeostatic processes than on-line fluctuations in cognitive content.

## Introduction

The existence of unceasing spontaneous brain activity has been recognized since at least the 1930s [1]. However, the functions of this activity have remained mysterious [2]. Over the last three decades, blood oxygen level dependent (BOLD) fMRI has become the dominant tool for measurement of brain activity in humans. Soon after the adoption of fMRI, it was observed that fMRI signals exhibit constant fluctuations unrelated to the task [3]. In the context of task fMRI, this activity was conventionally regarded as ‘physiological noise’ [4]. However, it is now clear that this ‘physiological noise’ is temporally correlated within functional systems [5,6]. It is this property of spontaneous brain activity that constitutes the basis of resting state functional connectivity (RSFC) [7]. The existence of this well-structured organization implies that spontaneous brain activity is physiologically consequential.

The meaning of spontaneous BOLD signal fluctuations has been variably interpreted along two different perspectives. According to one view, spontaneous BOLD fluctuations are proposed to reflect unconstrained cognitive processes, for example, retrospection, prospection, reflection, environmental monitoring — the ‘stream of consciousness’ — attendant to our subjective experience. Given the centrality of perception and action to mental life, it is appealing to assume that all observed brain activity is directly related to moment-to-moment cognition and behavior. This perspective has been reinforced by a massive accumulation of PET and fMRI experiments in which brain activity has been imaged with the objective of localizing cognitive operations [8]. More recently, the observation of ‘dynamic’ functional connectivity during wakeful rest and changes in functional connectivity between rest and task states have, at times, been interpreted as reflecting cognition [9,10].

We have previously articulated several problems with the notion that all ongoing BOLD activity directly reflects cognition and behavior (Box 1; [11]): (1) The topography of BOLD fMRI correlations remains largely intact during slow-wave sleep [12] and even anesthesia [13], states in which cognition is presumed to be either absent or greatly attenuated; (2) The extent to which task paradigms modify the correlation structure of spontaneous BOLD signal fluctuations is very limited [14,15,16^•^]; (3) While unconstrained cognition might be expected to vary from scan to scan within an individual, RSFC remains remarkably consistent across sessions [17,18]. RSFC is also relatively stable within a given scan, discounting fluctuations attributable to drowsiness [11] or arousal [19], which likely relate to fluctuations in BOLD signals, at least partly due to alterations in respiratory behavior and pCO2 [20]. Moreover, brain metabolic activity is high at all times and minimally affected by task performance [21].

For all of these reasons, unconstrained cognition does not fully explain ongoing spontaneous activity. An alternative view proposes that spontaneous BOLD activity may more closely relate to mechanisms associated with learning and memory [22^•^,^23^]. In the following, we review prior literature supporting the perspective that a substantial fraction of spontaneous brain activity represents homeostatic and consolidative signaling, the function of which is to enable neural plasticity while maintaining the brain's functional integrity through time. We also review recent evidence that BOLD RSFC may be intimately tied to these processes.

## Learning machine theory

When considering the role of ongoing neural activity in brain function, it is important to recognize that one of the brain’s primary capacities is its ability to learn new information about its environment. Theoretical considerations, initially formulated by David Marr [24], suggest that any associative learning machine functions optimally if it is allowed to alternate between two states: (1) a learning phase, during which the machine is connected to inputs and connections are enhanced between simultaneously active elements and (2) a restorative phase during which the machine is disconnected from inputs and connections between elements are rebalanced in a manner that increases randomness (entropy) [25,26^•^]. In multi-layer perceptrons, this principle is expressed as iterative alternation between a forward phase, during which prediction error is evaluated, and a backward phase, during which connection weights are adjusted by back-propagation [27]. The starkest expression of the state alternation principle in living organisms is sleep versus wake. This alternation appears to be necessary: all organisms capable of learning alternate between wake versus sleep states [28]. In vertebrates, events experienced during wake are registered in the hippocampus and the cerebral cortex [29,30]. During slow wave sleep (SWS), reactivation of the same circuits leads to the creation of stable (consolidated) episodic memory [31^•^].

Understanding how state alternation is implemented in brains requires consideration of the cellular and molecular events underlying synaptic weight modification. Activity-dependent synaptic plasticity plays a crucial role in brain development well before birth [32–34]. For example, retino-tectal connections have been shown to be sculpted by spontaneous retinal waves during prenatal development of the visual system [35]. Following birth, spontaneous activity continues to refine neural connections using sensory feedback [36,37]. During early life critical periods, experience-dependent synaptic plasticity tunes the response properties of cortical sensory neurons (e.g. ocular dominance columns) [38]. As the brain matures, metabolic ‘brakes’ limit neural plasticity to mechanisms centered on inhibitory interneurons [39–41]. Although neural plasticity in adults is more restricted, the underlying activity-dependent processes likely follow similar principles.

## Molecular mechanisms of activity-dependent synaptic plasticity

Activity-dependent synaptic plasticity is conventionally discussed under the headings of long-term potentiation (LTP) and long-term depression (LTD). But LTP/LTD are deceptively simple terms encompassing a wide range of molecular processes [42,43]. The early phase of LTP (E-LTP) is triggered by Ca^2+^ influx linked to post-synaptic depolarization, which sets in motion molecular cascades mediated by phosphorylation and dephosphorylation of regulatory molecules (e.g. protein kinase C (PKC) and Ca^2+^-calmodulin-dependent protein kinase (CamKII)) that govern neurotransmitter receptor trafficking. E-LTP lasts 1–3 hours and is independent of gene expression. The late phase of LTP (L-LTP) begins with the transcription of immediate early genes (IEGs; e.g. Arc, Zif268) that control translational processes, which lead, on a time scale of hours, to structural changes in dendritic spines [44–46]. Thus, whereas electrophysiological event-related responses may last up to a few hundred millisec and BOLD hemodynamic responses typically evolve over ~16–20 s, the metabolic traces of the evoked activity persist over much longer time scales. These traces may underlie the observation that fMRI responses to task A are modulated by having performed unrelated task B during the past half hour [47].

The Hebbian principle (‘fire together → wire together’) is often invoked to account for resting state functional connectivity [48,49]. The mechanism underlying Hebbian learning, that is, spike-timing dependent synaptic plasticity (STDP), has been elucidated in considerable detail [50,51]. In brief, neural back-propagation of depolarization induced by a first excitatory stimulus removes the Mg^2+^ block at NMDA receptors, thereby allowing a second stimulus (if it occurs within a 20–85 ms window) to induce local Ca^2+^ entry, which initiates the LTP molecular cascade, ultimately reinforcing the association between the paired stimuli. Hebbian mechanisms undoubtedly play a central role in adult learning. Accordingly, it is reasonable to posit that synchronous spontaneous BOLD fluctuations that give rise to RSFC are due to a history of prior co-activation. However, a system dominated by unopposed Hebbian plasticity inevitably becomes either infinitely active or silent.

In contrast to Hebbian plasticity, which adjusts synaptic weights in the same direction as an applied stimulus, the brain also employs various mechanisms of *homeostatic* plasticity, which adjusts synaptic strengths in the opposite direction to return excitatory/inhibitory (E/I) balance and mean firing rate to prior set points [52]. Homeostatic plasticity includes cell-autonomous mechanisms that directly adjust neuronal excitability to counteract environmental stimuli, as well as multiplicative synaptic scaling, which preserves relative strengths between neighboring synapses, thereby maintaining currently represented information [53^•^]. These homeostatic mechanisms operate at the level of dendritic branches [45], individual neurons [53^•^], and large-scale circuits [54], and are active over multiple time scales [55,56]. A correlate of these homeostatic processes is ongoing turnover of synaptic proteins and lipids with half-lives on the order of ‘minutes, hours, days, weeks’ [57]. Modeling experiments suggest that homeostatic regulation of E/I balance plays a crucial role in maintaining the characteristic features of spontaneous brain activity [58]. Importantly, synaptic homeostasis is inseparable from consolidation, the process whereby brief changes in neural activity ultimately lead to stable memory [59,60^•^]. Thus, it is reasonable to posit that spontaneous activity includes both Hebbian and homeostatic signaling.

## On-line versus off-line processes in electrophysiology

Consolidation characteristically takes place after the events and associated behavioral responses that will later be remembered. This defining feature motivates the distinction between on-line versus off-line processes. The consolidation of episodic memory through parahippocampal place-cell replay in association with hippocampal sharp-wave ripples (SWR), especially during SWS, exemplifies off-line processing [61,62]. Consolidation of procedural memory appears to be less dependent on SWS, but nevertheless is said to take place off-line [63,64]. In contrast, perception, motor behavior, retrospection, prospection, and rumination, all exemplify on-line processes. It is the central thesis of the present work that the distinction between on-line versus off-line processing in the brain is logically parallel to the state alternation principle discussed above in connection with theoretical and artificial learning machines. Thus, by analogy, we suggest that particular regions of the brain exist, at any given time, in a state dominated by either on-line or off-line processes.

Honey *et al.* have recently pointed out that the brain switches between externally oriented versus internally oriented modes at multiple temporal and spatial scales [65^•^]. This nomenclature differs from that used in the preceding discussion but we suggest that the distinction between external versus internal modes is closely related to, if not identical to, the distinction between on-line versus off-line processes. As noted above, wake versus sleep represents this distinction at the coarsest temporal and spatial scale. But neither wake nor sleep are homogeneous states. It is generally well recognized that sleep includes graded depth SWS as well as rapid eye movement (REM) stages. It probably is less well recognized that nominally awake subjects continually fluctuate between more versus less aroused states. In humans, this fluctuation manifests as variable task performance, changing EEG rhythms, and differing activity patterns as imaged with BOLD fMRI [19,66,67]. Similarly, awake rodents alternate between theta states, during which they actively explore their environment, versus quieter states during which theta is suppressed and SWRs occur [68–70]. In awake rodents, entorhinal cortex alternates between encoding versus retrieval modes depending on the phase of the theta rhythm [71]. Taking these considerations into account suggests that some portion of ongoing brain activity can be understood in terms of the two-phase learning machine principle manifesting as multiple, superimposed processes.

According to this model, each part of the brain alternates between on-line versus off-line states, and both states may be simultaneously present in different parts of the brain. Empirical evidence suggests that instantiation of the on-line state suppresses off-line activity locally. In the electrophysiology literature, this principle is known as stimulus quenching [72]. Probably the most robust illustration of stimulus quenching in fMRI is suppression of ongoing visual cortex BOLD signal fluctuations by eye opening [11,73]. It appears likely that ‘BOLD fluctuation quenching’ occurs in all parts of the cerebral cortex recruited by any task, although the magnitude of the effect may be modest [74]. BOLD fluctuation quenching is relevant to the observation that task performance modifies RS-FC (see below).

## Interpreting spontaneous BOLD fluctuations

It is well-established that evoked BOLD responses are linked to event-related neural activity [75,76]. The physiological links between resting state neural activity and BOLD signal fluctuations have been relatively less well studied [77,78], but the available evidence suggests that stimulus-evoked responses and spontaneous fluctuations in LFP amplitude are similarly coupled to BOLD signals [79,80]. A separate line of investigation suggests that infra-slow (<0.1 Hz) EEG potentials directly mirror resting state BOLD fMRI signal fluctuations [81,82]. Conceivably, metabotropic glutamatergic signaling may also be linked to BOLD fMRI fluctuations, especially as metabotropic glutamate receptors are thought to play an important role in neural homeostasis [83], although, as far as we are aware, direct evidence supporting such a link has not been reported.

What fraction of resting state BOLD fMRI signal fluctuations represents on-line versus off-line processes? Precise separation of these processes in humans is experimentally challenging and further confounded by artifact and other non-neural sources of variance in BOLD imaging [84,85]. However, numerous investigations of functional connectivity at rest and in various task states have provided significant insight. In general, it has been found that functional connectivity exhibits similar overall architecture regardless of state [14,15,86], albeit with specific measurable differences depending on task. Some investigators emphasize this latter observation, noting that it is possible to differentiate task states using FC [9,87]. Although this is true, Gratton *et al.* have shown that the effect of task state on FC is quantitatively minor in comparison to individual-specific and common group patterns of FC [16^•^]. One might expect that an externally imposed task would have a substantially greater impact on functional connectivity than concurrent task-independent thought. Therefore, these results suggest that functional connectivity differences between subjects likely do not represent task-independent cognition. Moreover, to the extent that there is task-related FC modulation, some portion may be explained by spatially specific suppression of off-line activity in brain areas recruited by the task, as previously discussed [74]. By contrast, task-evoked BOLD signals exhibit high dependence on task demands with similar patterns of recruitment across subjects [16^•^]. These findings support the notion that spontaneous and evoked BOLD signals likely reflect different underlying types of brain activity (i.e. off-line versus on-line).

This perspective is supported by a substantial literature on the effects of intensive task training on functional connectivity. This literature is premised on the idea that changes in functional connectivity reflect practice-related effects of Hebbian neural plasticity. Functional connectivity changes have been observed in the context of numerous training paradigms including visuomotor adaptation [88,89], Braille training in sighted individuals [90] visual perception practice [91], playing the Space Fortress videogame [92], and acquisition of episodic memory [93], among others [94].

Most recently, our laboratory has reported an experiment in which healthy participants had their dominant arm casted for two weeks [95^•^]. Extended resting state fMRI was acquired daily over the two weeks before, during, and after casting. This manipulation generated the largest within-subject RSFC changes of which we are aware. Specifically, motor cortex homotopic functional connectivity, which normally has a Pearson correlation ~0.7–0.8, was dramatically reduced (by as much as −0.86) within a few days of casting. The effect presumably reflects plasticity induced by the motor accommodations enforced by the cast, for example, having to use the non-dominant limb for habitual activities of daily living. Crucially, comparable RSFC changes were not induced by wearing the cast only during scanning. The implication is that the RSFC effect depends on *having had* the cast on during the past several hours to days. The time scale of this effect, that is, hours to days, strongly suggests that the mechanisms underlying the observed alterations in spontaneous activity are related to homeostatic and consolidative mechanisms as opposed to instantaneous cognitive content. As previously discussed, synaptic plasticity involves a multi-stage molecular cascade comprising 2^nd^ messenger signaling, gene transcription, and protein synthesis, which are expected to operate over a similar timescale.

In addition to large magnitude RSFC changes, casting led to increased amplitude of spontaneous fluctuations (ALFF) in the area undergoing plasticity and the emergence of spontaneous, large amplitude BOLD signal 'pulses’. The latter unexpected phenomenon may be a consequence of focal motor cortex disinhibition giving rise to paroxysmal activity [96]. By analogy with SWRs, these pulses may reflect a state of elevated neural plasticity induced by the extreme experimental manipulation [95^•^,^97^]. Importantly, the relative speed and magnitude of the casting effect suggests that RSFC can reflect recent experience to an extent greater than previously thought. The timecourse of the casting effect shows that it is possible to dramatically change RSFC in a matter of days, provided that the required adaptation to circumstances is of a sufficiently great magnitude. The anatomical specificity of the casting effect reflects the specificity of the behavioral constraint, which affected only the dominant upper extremity, but not the leg, face, or other functions involving activities of daily life. It should be emphasized that RSFC architecture normally is remarkably stable over short and long time-scales [11,16^•^,^18^]. Indeed, the spatial specificity of the casting effect demonstrates that the correlation structure of spontaneous BOLD signal fluctuations is largely preserved, even as regions affected by the manipulation exhibit change. Thus, spontaneous BOLD activity may reflect the results of long-term Hebbian-based functional organization, but may also reflect processes associated with neural plasticity itself.

## Future directions

Although the present arguments are circumstantial with regard to the physiological significance of spontaneous BOLD fluctuations, they are intended to provide a compelling framework for interpreting functional connectivity. Studies of RSFC, the effects of prior practice on RSFC, and the effects of concurrent task performance on functional connectivity may be most productively viewed from the perspective of overlapping on-line and off-line brain activity. In particular, manipulations of off-line activity (e.g. arousal, accumulated training effects, sensory deprivation, etc.) may be expected to impact FC at least as much as manipulations of cognitive content (though both may be expected to have a limited impact on an overall stable RSFC architecture). Substantial future work remains to validate this hypothesis and open questions remain. For instance, how does the time course of changes in BOLD fluctuations relate to structural, vascular, and metabolic manifestations of brain plasticity? Advanced concurrent imaging techniques, for example, qBOLD, DTI, VASO (see Ref. [94] for review) in the context of experiments inducing plasticity would provide significant insight along these lines. For example, measures of metabolic demand, for example, glycolytic index as measured by PET [98] and BOLD signal fluctuation amplitude [99] should be concurrently enhanced in areas undergoing plasticity. Further, animal models that connect BOLD signals with direct genetic or optogenetic manipulations of molecular mechanisms of synaptic modulation may close the loop on the causal relation between these measures. Thus, we may find that spontaneous brain activity indexed by ongoing BOLD fluctuations is more closely aligned with mechanisms of plasticity and synaptic homeostasis than ongoing cognitive content.

## Box 1 Evidence that RSFC structure is largely independent of cognitive content

1. RSFC structure is similar during wake and sleep. For instance, DMN structure is observed through wake, S1, S2, and SWS [12,100].
2. RSFC structure is present under anesthesia [13,101], although covariance does diminish with increased sedation [102].
3. RSFC structure is minimally altered by task state [14–16].
4. RSFC structure within subject is consistent across sessions [16,11,103].
5. RSFC structure within subject is similar over long time scales [18,17].
6. RSFC structure is consistent across subjects at the population level [104,105].
7. Similar RSFC structure is evident across mammalian species [106–108].
